# The Association Between Grip Strength and Depression Among Adults Aged 60 Years and Older: A Large-Scaled Population-Based Study From the Longitudinal Aging Study in India

**DOI:** 10.3389/fnagi.2022.937087

**Published:** 2022-06-24

**Authors:** Jinbao Wang, Xianghong Zhou, Shi Qiu, Linghui Deng, Jiakun Li, Lu Yang, Qiang Wei, Birong Dong

**Affiliations:** ^1^Department of Urology, West China Hospital, Sichuan University, Chengdu, China; ^2^West China School of Medicine, Sichuan University, Chengdu, China; ^3^National Clinical Research Center of Geriatrics, Center of Gerontology and Geriatrics, West China Hospital, Sichuan University, Chengdu, China

**Keywords:** depression, elderly, grip strength, LASI, risk factor

## Abstract

**Background:**

The association between grip strength and depression in elderly individuals in low- and middle-income countries (LMICs) has rarely been studied. This study aims to explore the relevance of grip strength and depression in the elderly population using data from a national large-scale population.

**Methods:**

This study was conducted using data from seniors over 60 years old in wave 1 of the Longitudinal Aging Study in India (LASI). Grip strength is the maximum of three measurements by the dynamometer. Depression symptoms were assessed using 10 items on the Center for Epidemiologic Studies Depression Scale (CESD-10) with a 10-point boundary. Multivariate linear regression analysis, non-linear analysis, subgroup analysis, interaction tests and sensitivity analysis were performed.

**Results:**

There were 27,343 participants in this study, including 19,861 participants with low grip strength and 7,482 participants with normal grip strength. The results revealed that grip strength and depression were negatively correlated in elderly individuals after adequate adjustment for confounding factors [odds ratio (OR) = 1.237, 95% confidence interval (CI) 1.172–1.305, *p* < 0.00001]. The results remained stable after adjusting for all confounding factors (OR = 1.090, 95% CI 1.030–1.155, *p* = 0.00307). Regression analysis showed that physical activity (PA), comorbidities and cognition may have an impact on the correlation between grip strength and depression symptoms. Smooth curve fit suggested that grip strength and depressive symptoms were linearly related. The interaction test results of gender in the relationship between grip strength and depression were significant (*p* for interaction < 0.05).

**Conclusion:**

Grip strength and depression were negatively correlated in older Indians, and larger prospective studies are needed in the future to determine this association.

## Introduction

Depression is an extremely important global health issue, threatening the health of an estimated 350 million people worldwide (McDowell et al., 2018), especially among elderly individuals. According to the World Health Organization (WHO), approximately 7% of the elderly population suffers from unipolar depression (World Health Organization Mental Health of Older Adults, 2017). Depression leads to decreased cognitive ability and quality of life (Kang et al., 2018) and increases the chances of chronic comorbidities such as diabetes, cardiovascular disease, and arthritis (Seo and Je, 2018; Vallerand et al., 2018; Xuan et al., 2018). People with depression have a higher risk of suicide (Chapman and Perry, 2008). Therefore, the prevention and treatment of depression is extremely important.

Grip strength is a measure of maximum static hand strength, which reflects overall muscle strength. Muscle strength is an important indicator for the evaluation of personal health status (Ihle et al., 2017) and is negatively related to the risk of death (Ruiz et al., 2008). Muscle strength will decline by 1.5–3.5% per year in seniors 60 and older (Kim and Choi, 2013). It has been well established that grip strength is positively related to the possibility of cardiovascular disease, neurological disease, metabolic syndrome, and disability in the future (McGrath et al., 2018). Grip strength has therefore been suggested as a biomarker for identifying older adults at risk for poor health (Bohannon, 2019). It is a non-invasive and reliable detection approach for muscle strength (Cooper et al., 2010). On the other hand, some studies believe that increased physical activity can reduce the risk of depressive symptoms (Hallgren et al., 2020). However, older adults may be less likely to participate in physical activity due to decreased muscle strength, and their risk of depression is increased accordingly (Schuch et al., 2018).

Numerous studies have investigated the correlation between grip strength and depression symptoms and have concluded that grip strength and depression are negatively correlated (Brooks et al., 2018; Lee et al., 2018; Marques et al., 2020). Some have also compared whether this relationship holds true for sex differences (McDowell et al., 2018), comorbidities of chronic disease (Lee, 2020), and cancer patients (Zhang et al., 2022). However, most of the participants in these studies were from developed countries, and there were relatively few studies on low- and middle-income countries (LMICs) (e.g., India). A previous study using data from six LMICs examined the relationship between grip strength and depression, arguing that lower grip strength points to a higher risk of depression in LMICs (Ashdown-Franks et al., 2019). However, the six countries included varied, and the sample size specific to each particular country was small.

To our knowledge, no studies have systematically applied national data to assess the association of grip strength and depression in elderly individuals in LMICs. As depression is more important in LMICs (Guerra et al., 2016), early identification of depression and effective interventions can alleviate symptoms, improve patients’ quality of life, and reduce social burden. Therefore, this study aims to explore the relevance of grip strength and depression in elderly individuals by using data from the Longitudinal Aging Study in India (LASI) and to gain a glimpse of the rules applicable to LMICs.

## Materials and Methods

### Data and Population

Our data were obtained from wave 1 of the LASI, which is India’s first study on aging, collecting data during 2017–2018 to understand the health, economic, social and psychological aspects of India’s aging process. For rural and urban areas, LASI employs a three- and four-stage sampling method, respectively, and ultimately 72,262 people over the age of 45 and their spouses were included in the survey.

**FIGURE 1:**
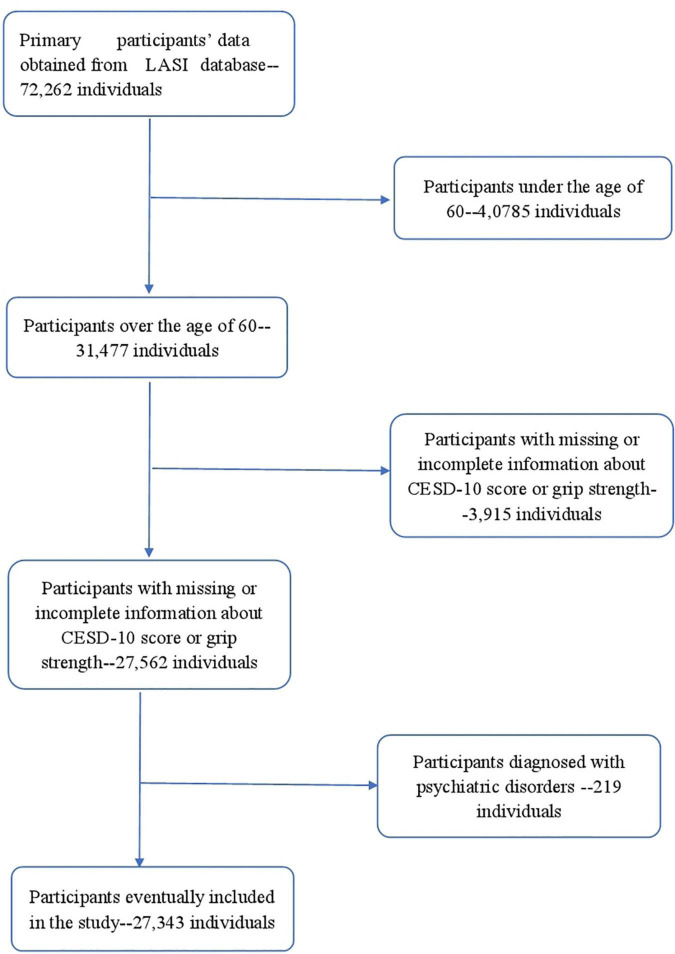
Flowchart of participant selection.

In this study, participants under the age of 60 without data on grip strength and depression scale were excluded, and individuals diagnosed with psychiatric disorders were also excluded; the remaining 27,343 participants were enrolled in the study (Figure 1). Each participant signed a written informed consent form, details of which can be found on the LASI wave 1 website.^1^

### Exposure and Outcome

Grip strength (kg) measured by a dynamometer was used as the exposure factor for this study. All participants followed the standard measurement procedure of grip strength, and squeezed the hand grip machine as hard as possible three times with 60 s intervals each time, and the maximum value was taken as the final grip strength of the participant. In this study, men with grip strength <30 kg and women <20 kg were defined as those with low grip strength (Cruz-Jentoft et al., 2010).

Depressive symptoms were the outcome and were obtained through a questionnaire. Participants’ depressive symptoms were determined using the 10-item Center for Epidemiologic Studies Depression Scale (CESD-10), which is an abridged version of the 20-item CESD that measures depressive symptoms in the general population (Boey, 1999). Respondents rated “How often have you felt this way in the past week” on a scale of 0–30. Participants were separated based on the CESD-10 score using a cutoff value of 10 for the presence or absence of depressive symptoms. Studies have shown that the predictive accuracy of CESD-10 scores ≥ 10 is no worse than that of 20-item Center for Epidemiologic Studies Depression Scale (CESD-20) scores with ≥ 16 as the cutoff (Andresen et al., 1994).

### Covariates

We extracted baseline characteristics of participants from the LASI database, including age, gender, BMI, education level, marital status, caste, and place of residence. Education was defined as never educated, middle school or under, secondary and higher secondary and above higher secondary. Married status was divided into married or partnered, widowed and others. Place of residence was classified by rural and urban areas. Regarding caste, India’s unique racial system, we divided it into scheduled caste, scheduled trible, other backward class and no or other caste. In addition, we extracted other potential confounders, such as history of smoking or drinking, history of hypertension, diabetes, chronic heart disease, pulmonary disease and arthritis. Annual per capita consumption expenditure was assessed using household consumption data. Physical activity (PA) was assessed by the question “How often do you take part in sports or vigorous activities, such as running or jogging, swimming, going to a health center or gym, cycling, digging with a spade or shovel, heavy lifting, chopping, farm work, fast cycling, cycling with loads (everyday, more than once a week, once a week, one to three times a month, hardly ever or never).” Then, we coded the physical activity frequency of “everyday” as frequent, “more than once a week, once a week, one to three times a month” as rare physical activity, and coded “hardly ever or never” as never (IIPS, 2020). Cognitive impairment was assessed using measures from the University of Michigan Health and Retirement Study (HRS), assessing five areas of memory, orientation, arithmetic function, executive function, and object naming. Memory was measured using immediate word recall and delayed word recall and scored 0–10; direction was measured by time and place and scored 0–4; backward counting (0–2), serial seven (0–5), computation method (0–2 points) to measure arithmetic function; paper folding (0–3) and pentagon drawing method (0–1) to measure executive function; object naming was assigned a score of 0–2 points. The final composite score was 0–43, with the lowest 10th percentile being considered to be cognitive impairment (India Report, 2020).

### Statistical Analysis

In the baseline characteristics, continuous variables are represented by means and standard deviations, while categorical variables are represented by rates and percentages. For continuous variables, *p*-values were obtained using the Kruskal–Wallis rank-sum test, and for categorical variables, if the theoretical number was <10, Fisher’s exact test was used. We used multivariate logistic regression analyses to explore the association between grip strength and CESD-10 scores, adjusting for different covariates to reduce the influence of other confounding factors in the study on the results. The first model was only adjusted for age and gender (Model I); the second model was adjusted for age, gender, education level, marital status, place of residence, caste and annual per capita consumption expenditure (Model II); the third model was adjusted for age, gender, education level, marital status, place of residence, caste, annual per capita consumption expenditure, drinking status, smoking status and physical activity (Model III); and the fully adjusted models were adjusted for age, gender, education level, marital status, place of residence, caste, annual per capita consumption expenditure, drinking status, smoking status, physical activity, comorbidities (diabetes, hypertension, chronic heart disease, pulmonary disease and arthritis) and cognitive impairment (Model IV). Subsequently, we used a generalized additive model (GAM) and smooth curve fitting to examine the non-linear correlation between grip strength and the risk of depression symptoms and further examined the correlation between grip strength and CESD-10 scores. To verify the accuracy and reliability of these associations, we performed subgroup analyses and interaction tests by variables (gender, BMI, education level, marital status, caste, annual per capita consumption expenditure, physical activity, and cognitive impairment). Offsets may occur due to non-standard coding of disease diagnoses. To reduce the impact of the exclusion of participants whose diagnoses shown in the database were psychiatric disorders but may actually be depression, we specifically performed a sensitivity analysis with the overall population that did not exclude other psychiatric disorders and constructed multiple regression models. The variables adjusted for the different models are the same as those performed in the regression analysis. All statistical results in this study were completed by R software, and a *p* value < 0.05 was considered to be statistically significant.

## Results

### Baseline Characteristics

There were 27,343 participants in this study, including 19,861 participants with low grip strength and 7,482 participants with normal grip strength. The average age of the study population was 68.615 ± 7.251 years, with 48.279% males and 51.721% females. Of the 27,343 participants, 53.337% had never been educated, 64.777% were married or had a partner, and 66.591% lived in rural areas. Supplementary Table 1 presents the baseline characteristics of the participants. Overall, people with low grip strength were more inclined to be older, have a lower BMI, have less PA, and have a greater chance of having arthritis. In addition, 12,377 (45.266%) participants had a CESD-10 score greater than 10, and the participants with low grip strength had a higher probability of CESD-10 scores greater than 10 (46.700% vs 41.460%). A total of 4,539 (16.600%) people were considered to be cognitively impaired, and elderly individuals with lower grip strength had a higher percentage of cognitive impairment than those with normal grip strength (19.400% vs 9.169%). Other information is presented in Supplementary Table 1.

### Association Between Grip Strength and Depression

Table 1 presents the results of multivariate logistic regression analyses of grip strength and depression symptoms. The results showed that grip strength was negatively correlated with depression symptoms; that is, elderly individuals with low grip strength had a higher risk of depression. The OR value of model 1 was 1.237 (95% CI 1.172–1.305, *p* < 0.00001) after adjusting for age and gender. This association remained stable after adjusting for multiple possible confounders. Even after adjusting for all the covariates included in model IV, the results were statistically significant (OR = 1.090, 95% CI 1.030–1.155, *p* = 0.00307). For the regression analysis, we can easily conclude from the results of Model I that gender and age may be risk factors affecting the relationship between grip strength and depression. From Model III, it is concluded that PA, drinking status and smoking status may play a role in this association. Model IV showed that comorbidities and cognition contributed to the relevance. When the CESD-10 score was analyzed as a continuous variable, even after adjusting for all variables that might have affected the results, Model IV showed a negative correlation between grip strength and CESD-10 scores (OR = 0.128, 95% CI 0.018–0.238, *p* = 0.02217).

**TABLE 1:** Multivariate regression model of the relationship between grip strength and the risk of depression.

Exposure	Crude model		Model I		Model II		Model III		Model IV	
	OR (95%CI)	*p*-value	OR (95%CI)	P-value	OR (95%CI)	P-value	OR (95%CI)	P-value	OR (95%CI)	P-value
Normal grip strength	Reference		Reference		Reference		Reference		Reference	
Low grip strength^a^	1.237 (1.172, 1.305)	<0.00001	1.193 (1.128, 1.261)	<0.00001	1.123 (1.061, 1.189)	0.00006	1.115 (1.053, 1.180)	0.00018	1.090 (1.030, 1.155)	0.00307
Low grip strength^b^	0.470 (0.362, 0.577)	<0.00001	0.351 (0.240, 0.461)	<0.00001	0.199 (0.089, 0.309)	0.00039	0.180 (0.070, 0.290)	0.0013	0.128 (0.018, 0.238)	0.02217

*^a^CESD score as categorical variable.*

*^b^CESD score as continuous variable.*

*Crude model adjust for none.*

*Model I adjust for: age; gender.*

*Model II adjust for: age; gender; education level; marital; place of residence; caste; annual per capita consumption expenditure.*

*Model III adjust for: age; gender; education level; marital; place of residence; caste; annual per capita consumption expenditure; drinking status; smoking status; physical activity.*

*Model IV adjust for: age; gender; education level; marital; place of residence; caste; annual per capita consumption expenditure; drinking status; smoking status; physical activity; diabetes; hypertension; chronic heart disease; pulmonary disease; arthritis; cognitive impairment.*

### Non-linear Correlation Analysis

**FIGURE 2:**
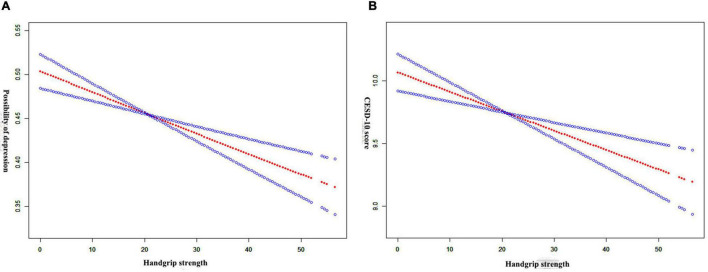
**(A)** GAM shows a linear association between grip strength and the possibility of depression symptoms; **(B)** GAM shows a linear association between grip strength and CESD-10 score.

The results of GAM and smooth curve fitting are presented in Figure 2A. We found that grip strength was negatively correlated with the risk of depression symptoms. Meanwhile, a consistent negative association between grip strength and CESD-10 scores was observed (Figure 2B).

### Subgroup Analysis

**FIGURE 3:**
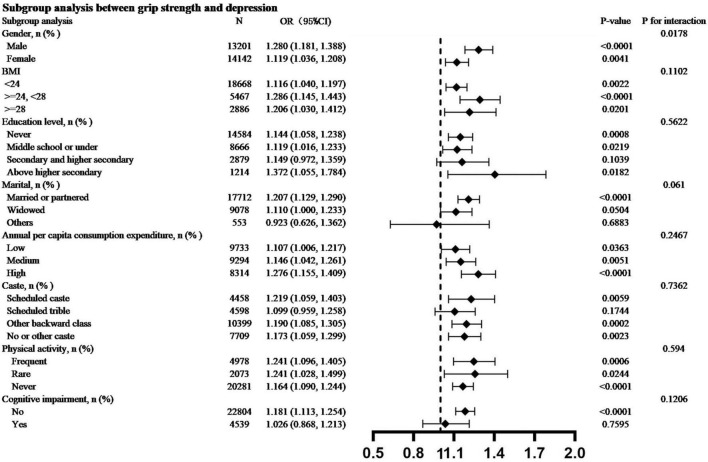
Subgroup analysis between low grip strength and depression.

Subgroup analyses indicated that lower grip strength was associated with a higher risk of depression (Figure 3), which is consistent with previous findings. Subgroup analysis of different variables showed that the negative correlation between grip strength and depression was stable. The results of the interaction test of gender showed significant differences (*p* values was 0.0178), indicating that gender plays important roles in the relationship between grip strength and depression. The P value of the interaction test for other variables is not statistically significant.

### Sensitivity Analysis

In the sensitivity analysis (Supplementary Table 2), 219 individuals whose diagnoses in the dataset were psychiatric disorders and had been excluded previously were included, and we performed a multiple logistic regression analysis on 27,343 participants. The results still showed a negative correlation between grip strength and depression and were stable and credible even after adjusting for all variables (Model IV OR = 1.092, 95% CI 1.031–1.156, *p* = 0.00255), which was consistent with the results of the previous multivariate logistic regression analyses and suggested that other psychiatric disorders are less disruptive to the stability of the relationship between grip strength and depression.

## Discussion

We explored the relationship between grip strength and depression in elderly individuals using data from LASI. To the best of our knowledge, this is the first large population-based study in recent years to focus on the association between grip strength and depression in older adults in LAMICs. In addition, we analyzed potential risk factors that may have an impact on the relationship between grip strength and depression, including gender, PA, comorbidities and cognition. Elderly individuals with lower grip strength tended to be at higher risk for depression, and even after adjusting for potential confounders, grip strength and depression showed a relatively stable negative correlation, and this association may be related to gender, PA, comorbidities, and cognition.

A study explored the correlation between grip strength and depression in elderly individuals in six LAMICs (Ashdown-Franks et al., 2019), including India. However, the study used data from SAGE^2^ a decade ago and ultimately did not find any gender differences in the association between low grip strength and depressive symptoms. A 7-year prospective cohort study in China sought to investigate whether insufficient muscle strength contributes to a higher incidence of depression (Bao et al., 2022). This study, with participants who were middle-aged and elderly people over the age of 45, was a small sample study, and the influence of confounding factors such as gender and exercise were ignored. Grip strength and depression have been shown to be negatively correlated in older adults, and the correlation is stronger in females (McDowell et al., 2018). They believe this may be due to the relatively low incidence of depression in males, and argue that it is more necessary for women than men to maintain a good level of physical strength later in life (Veronese et al., 2017). Resistance training can reduce depressive symptoms in older adults (Ansai and Rebelatto, 2015). Regular physical activity, including aerobic exercise and muscle-building exercises, may be the best option for preventing depression (Bennie et al., 2020). This may be because higher muscle strength levels are associated with greater physical activity, and grip strength is a reliable indicator of muscle strength (Cooper et al., 2010). This is consistent with the findings of our study. A study using data from 12 European countries participating in the SHARE (European Survey on Health, Aging and Retirement) panel suggested that sudden onset of depressive symptoms may be associated with successive episodes of hypotonia (Bertoni et al., 2018). Depression research for LAMICs is limited, however, the incidence of depression is increasing rapidly in these countries. Our study population was large, and focused on older adults in LAMICs.

The negative correlation between grip strength and depression is the most important conclusion of our study. In addition, the effects of gender, PA, systemic inflammatory chronic diseases and cognition on the relationship between grip strength and depressive symptoms cannot be ignored. The specific mechanism can be understood from the following aspects. The temperament present in infancy and early childhood, cognitive vulnerability-stress interactions, and the role of puberty and timing of puberty may have contributed to the higher incidence of depression in females than in males (Hyde et al., 2008). Therefore, the potential importance of maintaining a higher grip strength level in later life is more important for females than males (Veronese et al., 2017).

Although the interaction test for physical activity did not show a significant difference, the people with low grip strength were more inclined to be less PA, and multivariate logistic regression analyses revealed that PA may be one of the risk factors. Depression leads to a decline in physical function through the influence of poor health behaviors (Penninx et al., 2000), which hinders the completion of daily living tasks in the elderly and reduces the sense of achievement in the elderly (Laredo-Aguilera et al., 2018). At the same time, declines in functional performance in older adults can lead to reduced activities of daily living and ultimately increase the risk of depression (Yanagita et al., 2006). From a physiological mechanism, muscle contraction is achieved through the release of cytokines and myokines, and myokines can reduce the risk of depression (Köhler et al., 2017). Previous meta-analyses have demonstrated that moderate physical activity may reduce inflammatory markers (including CRP, IL-6, etc.) (Lin et al., 2015). Since PA is helpful in preventing depression in older adults, the WHO recommends that older adults over 65 should engage in an equivalent combination of moderate- and vigorous-intensity activity weekly to obtain health benefits (Who Guidelines Approved by the Guidelines Review Committee, 2020).

Our study found an effect of chronic systemic inflammatory disease on the relationship between grip strength and depression. Systemic inflammatory diseases, such as cardiovascular disease, diabetes and rheumatoid arthritis (Rutledge et al., 2006; Mezuk et al., 2008; van’t Land et al., 2010), often coexist with depression, and increased peripheral inflammation may lead to sarcopenia (Bano et al., 2017), which is closely related to the mechanism of depression (Bertoni et al., 2018). Some studies have shown that inflammatory factors such as C reactive protein (CRP) (Bano et al., 2017), interleukin (IL)-6 (Belizário et al., 2016), and tumor necrosis factor alpha (TNF-α) (Powers et al., 2016) can lead to muscle atrophy or decreased muscle strength. In addition, proinflammatory cytokines such as IL-1, IL-6, TNF-α, and interferon (IFN)-γ enter the brain through multiple pathways to mediate depression-related psychiatric symptoms (Banks et al., 1995; Dantzer, 2001). Peripheral cytokines alter neurotransmitter function by activating local central nervous system (CNS) inflammatory networks (Felger and Lotrich, 2013). Anti-inflammatory and antioxidant substances provided through a healthy diet may reduce the damage caused by inflammatory substances. Studies have shown that healthy dietary patterns are positively associated with grip strength (Kim and Kwon, 2019; Zhang et al., 2021).

Our study found that cognition also had an impact on the relationship between grip strength and depression; in particular, older adults with low grip strength are at higher risk for cognitive impairment. A cross-sectional study of a population-scale dataset of 110,067 people is the first to suggest that grip strength can be a marker of cognitive function in patients with major depressive disorder or bipolar disorder (Firth et al., 2018). A meta-analysis of 48 voxel-based morphometry (VBM) studies demonstrated that patients with major depressive disorder and mild cognitive impairment were associated with a greater volume reduction in multiple regions of the brain (such as the insula, superior temporal gyrus and inferior frontal gyrus) (Zacková et al., 2021), which may be helpful in understanding this correlation. As we know, with age, cognitive decline leads to a decline in physical function (Demnitz et al., 2016). As previously discussed, decreased physical function may be accompanied by decreased PA and decreased muscle strength, thereby exacerbating the risk of depression. Else, as mentioned earlier, patients with depression tend to have higher levels of inflammatory factors in their bodies, and individuals with elevated inflammation are more likely to develop cognitive impairment. Else, as mentioned earlier, patients with depression tend to have higher levels of inflammatory factors, and individuals with elevated inflammation are more likely to observe cognitive impairment, and the two may rely on inflammatory factors to interact (Rosenblat et al., 2014).

Furthermore, researchers hold the view that this association between grip strength and depression may be related to decreased hippocampal volume (HCV) and white matter hyperintensity (WMH) (Firth et al., 2020). A study of older adults in England attempted to explore the association among grip strength and depression and chronic stress utilizing hair cortisol concentrations but was inconclusive (Smith et al., 2019).

The study has great advantages. To our knowledge, this is the first study to use nationwide data from LASI to investigate the association between grip strength and depression; meanwhile, this is the first study in recent years to do so with elderly individuals in LAMICs. This study found that grip strength and depression were negatively correlated in older adults, which can be extended from India to other LAMICs. Given that LAMICs carry a greater burden of depression, accounting for more than 80% of total years of disability globally (World Health Organization, 2017), these disorders account for less than 2% of the health budget in these countries (Jacob et al., 2007). Therefore, special attention is required for the grip strength level of elderly individuals so that the occurrence of depressive symptoms can be prevented and blocked at an early stage. However, this study also has some limitations. First, this study is a cross-sectional study and no firm conclusions can be drawn about the orientation on the relationship between grip strength and depression. Second, the samples in this study come from a single country, and the conclusions drawn may not be applicable in other countries and regions due to regional and cultural differences. Furthermore, as the data from LASI on dietary and inflammatory markers are missing, their contribution to the association of grip strength and depression could not be assessed. In the future, larger and more comprehensive prospective studies are warranted to verify our findings. Nonetheless, this study contributes to the association between grip strength and depression in older adults in LAMICs.

## Conclusion

Our study indicates that grip strength and depression were negatively correlated in older Indians, and gender, PA, systemic inflammatory chronic diseases and cognition all play a role in this correlation. Therefore, it is extremely important to take measures to improve grip strength and thus reduce the incidence of depressive symptoms in older adults, especially in LAMICs. More large prospective studies are warranted to verify the results in the future.

## Data Availability Statement

Publicly available datasets were analyzed in this study. This data can be found here: https://www.iipsindia.ac.in/content/lasi-wave-i.

## Ethics Statement

The studies involving human participants were reviewed and approved by Indian Council of Medical Research and all collaborating institutions. The patients/participants provided their written informed consent to participate in this study.

## Author Contributions

QW and BD presented this research. JW, XZ, and SQ participated in collecting, and analyzing the data and writing the manuscript. JL participated in some data-related work. LY and LD were mainly involved in some data analyses. All authors have considered and finally completed this manuscript.

## Conflict of Interest

The authors declare that the research was conducted in the absence of any commercial or financial relationships that could be construed as a potential conflict of interest.

## Publisher’s Note

All claims expressed in this article are solely those of the authors and do not necessarily represent those of their affiliated organizations, or those of the publisher, the editors and the reviewers. Any product that may be evaluated in this article, or claim that may be made by its manufacturer, is not guaranteed or endorsed by the publisher.

## Footnotes

1
https://www.iipsindia.ac.in/sites/default/files/LASI_India_Report_2020_compressed.pdf

2
http://www.who.int/healthinfo/sage/en/

## Abbreviations

OR: odds ratio
CI: confidence interval
WHO: World Health Organization
LMICs: low- and middle-income countries
LASI: Longitudinal Aging Study in India
CESD-10: 10-item Center for Epidemiologic Studies Depression Scale
PA: physical activity
CRP: C reactive protein
IL: interleukin
TNF- α: tumor necrosis factor alpha
IFN: interferon
CNS: central nervous system
HCV: hippocampal volume
WMH: white matter hyperintensity
GAM: generalized additive model
VBM: voxel-based morphometry
CESD-20: 20-item Center for Epidemiologic Studies Depression Scale.
