# Supplementary material for: The Association Between Grip Strength and Depression Among Adults Aged 60 Years and Older: A Large-Scaled Population-Based Study From the Longitudinal Aging Study in India
Source: Front Aging Neurosci. 2022 Jun 24;14:937087. doi: 10.3389/fnagi.2022.937087 (PMC9269105; doi:10.3389/fnagi.2022.937087)
Supplement: Supplementary file 1 [file Table_1.docx]

| **Supplement 1 Baseline characteristics of the participants** | | | | | |
| --- | --- | --- | --- | --- | --- |
| Variables | Total | Normal grip strength | Low grip strength | Standardize diff | P-value |
| N | 27343 | 7482 | 19861 |  |  |
| Age (year) (mean, SD) | 68.615 ±7.251 | 65.695 ±5.270 | 69.715±7.583 | 0.616 (0.589, 0.643) | <0.001 |
| BMI (mean, SD) | 22.126 ±4.655 | 23.419 ±4.446 | 21.633±4.638 | 0.393 (0.366, 0.420) | <0.001 |
| Grip strength (kg) (mean, SD) | 20.786 ±7.654 | 28.412±6.534 | 17.914±5.866 | 1.691 (1.661, 1.721) | <0.001 |
| CESD-10 score, n (%) |  |  |  | 0.106 (0.079, 0.132) | <0.001 |
| <10 | 14966 (54.734%) | 4380 (58.540%) | 10586 (53.300%) |  |  |
| >=10 | 12377 (45.266%) | 3102 (41.460%) | 9275 (46.700%) |  |  |
| Gender, n (%) |  |  |  | 0.009 (-0.017, 0.036) | 0.493 |
| Male | 13201 (48.279%) | 3587 (47.942%) | 9614 (48.406%) |  |  |
| Female | 14142 (51.721%) | 3895 (52.058%) | 10247 (51.594%) |  |  |
| Education level, n (%) |  |  |  | 0.218 (0.191, 0.244) | <0.001 |
| Never | 14584 (53.337%) | 3460 (46.244%) | 11124 (56.009%) |  |  |
| Middle school or under | 8666 (31.694%) | 2576 (34.429%) | 6090 (30.663%) |  |  |
| Secondary and higher secondary | 2879 (10.529%) | 971 (12.978%) | 1908 (9.607%) |  |  |
| Above higher secondary | 1214 (4.440%) | 475 (6.349%) | 739 (3.721%) |  |  |
| Marital, n (%) |  |  |  | 0.246 (0.219, 0.272) | <0.001 |
| Married or partnered | 17712 (64.777%) | 5458 (72.948%) | 12254 (61.699%) |  |  |
| Widowed | 9078 (33.200%) | 1877 (25.087%) | 7201 (36.257%) |  |  |
| Others | 553 (2.022%) | 147 (1.965%) | 406 (2.044%) |  |  |
| Smoking status, n (%) |  |  |  | 0.034 (0.007, 0.060) | 0.05 |
| Never | 21724 (79.488%) | 5947 (79.548%) | 15777 (79.465%) |  |  |
| Current | 3884 (14.211%) | 1097 (14.674%) | 2787 (14.037%) |  |  |
| Ever | 1722 (6.301%) | 432 (5.778%) | 1290 (6.497%) |  |  |
| Drinking status, n (%) |  |  |  | 0.012 (-0.014, 0.039) | 0.67 |
| Never | 22566 (82.554%) | 6200 (82.888%) | 16366 (82.428%) |  |  |
| Current | 2453 (8.974%) | 659 (8.810%) | 1794 (9.036%) |  |  |
| Ever | 2316 (8.473%) | 621 (8.302%) | 1695 (8.537%) |  |  |
| Place of residence, n (%) |  |  |  | 0.050 (0.024, 0.077) | <0.001 |
| Urban | 9135 (33.409%) | 2629 (35.138%) | 6506 (32.758%) |  |  |
| Rural | 18208 (66.591%) | 4853 (64.862%) | 13355 (67.242%) |  |  |
| Caste, n (%) |  |  |  | 0.155 (0.129, 0.182) | <0.001 |
| Scheduled caste | 4458 (16.411%) | 1089 (14.698%) | 3369 (17.054%) |  |  |
| Scheduled trible | 4598 (16.927%) | 1291 (17.425%) | 3307 (16.740%) |  |  |
| Other backward class | 10399 (38.282%) | 2578 (34.796%) | 7821 (39.590%) |  |  |
| No or other caste | 7709 (28.379%) | 2451 (33.081%) | 5258 (26.616%) |  |  |
| Annual per capita consumption expenditure, n (%) |  |  |  | 0.125 (0.098, 0.152) | <0.001 |
| Low | 9733 (35.599%) | 2387 (31.903%) | 7346 (36.991%) |  |  |
| Medium | 9294 (33.993%) | 2546 (34.028%) | 6748 (33.980%) |  |  |
| High | 8314 (30.409%) | 2549 (34.068%) | 5765 (29.030%) |  |  |
| Hypertension, n (%) |  |  |  | 0.008 (-0.019, 0.034) | 0.58 |
| No | 17907 (65.500%) | 4880 (65.241%) | 13027 (65.597%) |  |  |
| Yes | 9432 (34.500%) | 2600 (34.759%) | 6832 (34.403%) |  |  |
| Diabetes, n (%) |  |  |  | 0.002 (-0.025, 0.028) | 0.91 |
| No | 23133 (84.612%) | 6326 (84.572%) | 16807 (84.627%) |  |  |
| Yes | 4207 (15.388%) | 1154 (15.428%) | 3053 (15.373%) |  |  |
| Chronic heart disease, n (%) |  |  |  | 0.015 (-0.012, 0.042) | 0.273 |
| No | 25973 (94.993%) | 7125 (95.229%) | 18848 (94.904%) |  |  |
| Yes | 1369 (5.007%) | 357 (4.771%) | 1012 (5.096%) |  |  |
| Arthritis, n (%) |  |  |  | 0.096 (0.069, 0.122) | <0.001 |
| No | 23544 (86.201%) | 6617 (88.545%) | 16927 (85.318%) |  |  |
| Yes | 3769 (13.799%) | 856 (11.455%) | 2913 (14.682%) |  |  |
| Pulmonary disease, n (%) |  |  |  | 0.023 (-0.004, 0.049) | 0.1 |
| No | 26609 (97.319%) | 7301 (97.581%) | 19308 (97.221%) |  |  |
| Yes | 733 (2.681%) | 181 (2.419%) | 552 (2.779%) |  |  |
| Physical activity, n (%) |  |  |  | 0.176 (0.149, 0.202) | <0.001 |
| Frequent | 4978 (18.213%) | 1678 (22.430%) | 3300 (16.624%) |  |  |
| Rare | 2073 (7.585%) | 676 (9.036%) | 1397 (7.037%) |  |  |
| Never | 20281 (74.202%) | 5127 (68.534%) | 15154 (76.339%) |  |  |
| Cognitive impairment, n (%) |  |  |  | 0.296 (0.269, 0.322) | <0.001 |
| No | 22804 (83.400%) | 6796 (90.831%) | 16008 (80.600%) |  |  |
| Yes | 4539 (16.600%) | 686 (9.169%) | 3853 (19.400%) |  |  |
| Note: BMI, Body mass index; CESD, Center for Epidemiologic Studies Depression Scale. | | | |  |  |
