# Supplementary material for: The Association Between Grip Strength and Depression Among Adults Aged 60 Years and Older: A Large-Scaled Population-Based Study From the Longitudinal Aging Study in India
Source: Front Aging Neurosci. 2022 Jun 24;14:937087. doi: 10.3389/fnagi.2022.937087 (PMC9269105; doi:10.3389/fnagi.2022.937087)
Supplement: Supplementary file 2 [file Table_2.docx]

| **Supplement 2 Multivariate regression model of the relationship between grip strength and the risk of depression--Sensitivity analysis** | | | | | | | | | | |
| --- | --- | --- | --- | --- | --- | --- | --- | --- | --- | --- |
| Exposure | Crude model | | Model I | | Model II | | Model III | | Model IV | |
|  | OR (95%CI) | P-value | OR (95%CI) | P-value | OR (95%CI) | P-value | OR (95%CI) | P-value | OR (95%CI) | P-value |
| Normal grip strength | Reference |  | Reference |  | Reference |  | Reference |  | Reference |  |
| Low grip strength^a^ | 1.239 (1.175, 1.307) | <0.00001 | 1.195 (1.130, 1.263) | <0.00001 | 1.125 (1.064, 1.191) | 0.00006 | 1.117 (1.056, 1.183) | 0.00012 | 1.092 (1.031, 1.156) | 0.00255 |
| Low grip strength^b^ | 0.471 (0.364, 0.578) | <0.00001 | 0.353 (0.242, 0.463) | <0.00001 | 0.202 (0.092, 0.312) | 0.00039 | 0.182 (0.072, 0.292) | 0.00117 | 0.128 (0.018, 0.237) | 0.0224 |
| ^a^CESD score as categorical variable; ^b^CESD score as continuous variable | | | | |  |  |  |  |  |  |
| Crude model adjust for none； | |  |  |  |  |  |  |  |  |  |
| Model I adjust for: age; gender | |  |  |  |  |  |  |  |  |  |
| Model II adjust for: age; gender; education level; marital; place of residence; caste; annual per capita consumption expenditure | | | | | | | |  |  |  |
| Model III adjust for: age; gender; education level; marital; place of residence; caste; annual per capita consumption expenditure; drinking status; smoking status; physical activity | | | | | | | | | | |
| Model IV adjust for: age; gender; education level; marital; place of residence; caste; annual per capita consumption expenditure; drinking status; smoking status; physical activity; diabetes; hypertension; chronic heart disease; pulmonary disease; arthritis; cognitive impairment | | | | | | | | | | |
